# The Evolving Definition of Education Scholarship: What the Clinician Educator Needs to Know

**DOI:** 10.5811/westjem.2016.12.33326

**Published:** 2016-12-15

**Authors:** Douglas S. Ander, Jeffrey N. Love

**Affiliations:** * Emory University School of Medicine, Department of Emergency Medicine, Atlanta, Georgia; † Georgetown University School of Medicine, Department of Emergency Medicine, Washington, D.C.

Medical education is faced with a growing number of challenges. The playing field that most of us know and recognize has been evolving over the past decade. Many of the truths we knew as educators are no longer accurate and we are faced with educating our learners in this new environment. Accreditation standards through national organizations are more rigorous and based on attainment of competency; therefore, outcome-based education has developed as a key factor. The Accreditation Council for Graduate Medical Education (ACGME) introduced the six domains of clinical competency to the profession, and in 2009 it began a multiyear process of restructuring its accreditation system to be based on educational outcomes in these competencies.^1^ The Liaison Committee on Medical Education in standard 6.1 of its Functions and Structure of a Medical School states that “the faculty of a medical school define its medical education program objectives in outcome-based terms that allow the assessment of medical students’ progress in developing the competencies that the profession and the public expect of a physician.”^2^ Both undergraduate and graduate medical education accreditation agencies are focusing on educational outcomes. It is no longer good enough to demonstrate that your learners performed the skills; now you must document achievement of those competencies. Our clinical environment is less conducive to concentrating on education due to documentation, billing requirements, and the sheer volume in our emergency room departments.^3^–^4^ Evolving educational pedagogy is more focused on small groups, simulation, and less on large-group formats. These challenges are opportunities for educators but require new strategies, which require research to determine the best approach.

The *Western Journal of Emergency Medicine* (*West*JEM) dedicated itself two years ago to being a forum for educational scholarship. Partnering with the Council of Emergency Medicine Residency Directors (CORD) and the Clerkship Directors in Emergency Medicine (CDEM), *West*JEM has developed an education supplement whose goal is to promote quality educational scholarship. Every educator, as a necessary element of their regular responsibilities, should generate educational scholarship. All quality teaching is based on a scholarly approach that will naturally lead to educational scholarship.

The definition of scholarship has evolved over the past several decades. In 1990 with the release of the Boyer report for the Carnegie Foundation a clearer definition of scholarship was defined.^5^ Boyer described four types of scholarship: discovery, integration, application, and teaching (Table). Discovery is what we typically consider to be traditional research, using the scientific method to objectively investigate the phenomenon under study. Integration interprets the use of knowledge across disciplines. An educator reports whether their experiences are useful beyond their own discipline. The third element, Application, focuses on using the educator’s findings to aid society. The final type of scholarship is Teaching, when a scholarly approach is used as the basis for teaching. This means studying various teaching models and practices to optimize learning.

Glassik in 2000 expanded on Boyer’s work by defining how we should measure quality in scholarship.^6^ He stated that for scholarship to be praised it must be characterized by clear goals, adequate preparation, appropriate methods, outstanding results, effective communication, and a reflective critique. It is vitally important that the distinction between teaching and scholarly teaching be clear. That distinction was clarified by Shulman when he stated that scholarly work must meet these criteria:

- The work must be made public.
- The work must be available for peer review and critique according to accepted standards.
- The work must be able to be reproduced and built on by other scholars.^7^

Educators must keep in mind that any teaching can be considered scholarship if the endeavor is approached systematically and proactively in a scholarly manner. The teaching that is done on a regular basis or new curricula may be studied as research (Scholarship of Discovery) or approached as educational scholarship (Scholarship of Teaching). The key is applying Glassick’s standards for scholarship and determining whether the test of scholarship proposed by Shulman is demonstrated in the work you are pursuing. Crites et al state that “The educational discovery (research) and teaching scholarly traditions are based upon different assumptions and utilize different methods, but they address similar educational questions and goals and are equally important for the development of educational scholars.” Their article in Medical Teacher provides a good introductory guide to education scholarship, its definitions, and practical advice on how to be successful.^8^

Most recently, a Consensus Conference on Educational Scholarship was convened by the AAMC-GEA in 2006.^9^ One aim of the conference was to reaffirm a previously identified group of five educational activity categories commonly identified as scholarship within educators’ portfolios, beyond education research. Through an iterative process, conference participants developed standards for these five educational activities consistent with principles of scholarship with the goal of facilitating the ability of promotion committees to evaluate the value of educators’ contributions. The five educational activities include the following:

- Teaching
- Curriculum development
- Advising and mentoring
- Education leadership and administration
- Learner assessment

The documentation standard for each category consists of two components: (1) Educational excellence in terms of quantity and quality; and (2) Engagement with the education community by documenting how the educators’ work was informed by current knowledge in the field.

The goal for educators is turning their educational programming and responsibilities into educational scholarship. The first step is to develop a working understanding of the subject at hand by reviewing the available literature. This provides a conceptual framework for the work that follows.^10^ One particularly useful approach is to consider the following three-phase model. Phase 1: Clearly describe what you want to do. What is the educational activity? Phase 2: Collect data to improve what you do or prove your hypothesis. This is the scholarly approach aspect. Finally, Phase 3: Share your finding to improve what the rest of the community does. This is scholarship. We propose that all educators apply these principles to all their teaching endeavors.

Our challenges provide us with the opportunity to be innovative and apply the scholarly approach as we tackle our new educational environment. When approached using Glassick’s definitions of scholarship and if they meet the test of true scholarship you will be adding to the greater body of literature that will improve our learners’ educational experience. We encourage everyone to consider approaching all their educational programs using a scholarly approach. Every time you are teaching or developing a curriculum be scholarly; not only will the result be pedagogically sound it will also be a basis for educational scholarship.

## Figures and Tables

**Table:** Boyer’s classification of the four types of scholarship.

Type of scholarship	Description
Scholarship of discovery	Original research
Scholarship of integration	Making connections across disciplines
Scholarship of application	Use of research, experience and expertise to provide a service to the greater community
Scholarship of teaching	Study of teaching and learning processes in a systematic method to optimize learning
